# Supplementary material for: Consensus for Flow Cytometry Clinical Report on Multiple Myeloma: A Multicenter Harmonization Process Merging Laboratory Experience and Clinical Needs
Source: Cancers (Basel). 2023 Mar 30;15(7):2060. doi: 10.3390/cancers15072060 (PMC10093543; doi:10.3390/cancers15072060)
Supplement: Supplementary file 1 [file cancers-15-02060-s001.zip › cancers-2190165-supplementary.pdf]

Consensus for flow cytometry clinical report on multiple myeloma: an Italian multicenter experience of harmonization.

Iole Cordone[1*], Rachele Amodeo[2], Silvia Bellesi[3], Fiorella Bottan[4], Francesco Buccisano[5], Maria Stefania De Propris[6], Serena Masi[1], Valentina Panichi[7], Maria Cristina Scerpa[8], Ombretta Annibali[9], Velia Bongarzoni[10], Tommaso Caravita di Toritto[11], Ugo Coppetelli[8], Luca Cupelli[12], Luca Franceschini[5], Mariagrazia Garzia[13], Alessia Fiorini[14], Giacinto Laverde[15], Andrea Mengarelli[16], Tommaso Za[3] and Maria Teresa Petrucci[17].

[1] Department of Research, Advanced Diagnostic and Technological Innovation, IRCCS Regina Elena National Cancer Institute, Rome, Italy

[2] Clinical Pathology and Biochemistry, Sant'Andrea University Hospital, Rome, Italy

[3] Department of Diagnostic Imaging, Oncological Radiotherapy and Haematology, IRCCS Foundation A. Gemelli University Hospital, Rome, Italy

[4] Clinical Pathology, San Giovanni Addolorata Hospital, Rome, Italy

[5] Department of Biomedicine and Prevention, Haematology and Stem Cell Transplant Unit University of Rome 'Tor Vergata', Rome, Italy

[6] Department of Translational and Precision Medicine, Immunophenotype Laboratory, 'Sapienza' University, Rome, Italy

[7] Department of Oncology and Haematology, Microbiology and Virology Unit, Belcolle Central Hospital, Viterbo, Italy

[8] Haematology and Stem Cell Unit, Santa Maria Goretti Hospital, ASL Latina, Italy

[9] Haematology and Stem Cell Transplant Unit, Campus Bio-Medico University, Roma, Italy

[10] San Giovanni Addolorata Hospital, Haematology, Rome, Italy

[11] Haematology, ASL Roma 1, Rome, Italy

[12] Haematology, Sant'Eugenio Hospital, ASL Roma 2, Rome, Italy

[13] Haematology and Stem Cell Transplant Unit, San Camillo Forlanini Hospital, Rome, Italy

[14] Department of Oncology and Haematology, Belcolle Central Hospital, Viterbo, Italy

[15] Haematology Unit, Sant'Andrea University Hospital, Rome, Italy

[16] Department of Research and Clinical Oncology, IRCCS Regina Elena National Cancer Institute, Rome, Italy

[17] Department of Translational and Precision Medicine, Haematology, 'Sapienza' University, Rome, Italy

KEYWORDS

multiple myeloma, flow cytometry, clinical report, MRD

CORRESPONDING AUTHOR *

Iole Cordone

Clinical Pathology and Cancer Biobank

Department of Research, Advanced Diagnostics and Technological Innovation

IRCCS Regina Elena National Cancer Institute

Via E. Chianesi 53, 00144, Rome, Italy

Tel. +39 06 52665110

e-mail: iole.cordone@ifo.it

SUPPLEMENTARY TABLE

Table S1. Flow cytometry diagnostic laboratories and hematological clinical centers participating to the study.

Flow cytometry laboratories	Clinical Haematology Units
IRCCS Regina Elena National Cancer Institute<br>Department of Research, Advanced Diagnostics and Technological Innovation<br>Clinical Pathology and Cancer Biobank<br>Rome, Italy	IRCCS Regina Elena National Cancer Institute<br>Department of Research and Clinical Oncology<br>Haematology Unit<br>Rome,
Sant'Andrea University Hospital<br>Clinical Pathology and Biochemistry<br>Rome, Italy	Sant'Andrea University Hospital<br>Haematology Unit<br>Rome, Italy
IRCCS Foundation A. Gemelli University Hospital,<br>Department of Diagnostic Imaging, Oncological Radiotherapy and Haematology<br>Rome, Italy	IRCCS Foundation A. Gemelli University Hospital,<br>Department of Diagnostic Imaging, Oncological Radiotherapy and Haematology<br>Rome, Italy
San Giovanni Addolorata Hospital<br>Clinical Pathology<br>Rome, Italy	San Giovanni Addolorata Hospital<br>Haematology<br>Rome, Italy
University of Rome 'Tor Vergata'<br>Department of Biomedicine and Prevention<br>Rome, Italy	University of Rome 'Tor Vergata'<br>Haematology - Stem Cell Transplant Unit<br>Rome, Italy
'Sapienza' University of Rome<br>Department of Translational and Precision Medicine<br>Immunophenotype laboratory<br>Rome Italy	'Sapienza' University of Rome<br>Department of Translational and Precision Medicine<br>Heamatology<br>Rome, Italy
Belcolle Central Hospital<br>Department of Oncology and Haematology<br>Microbiology and Virology Unit<br>Viterbo, Italy	Belcolle Central Hospital<br>Department of Oncology and Haematology<br>Viterbo, Italy
Santa Maria Goretti Hospital<br>Haematology and Stem Cell Unit<br>ASL Latina, Italy	Santa Maria Goretti Hospital<br>Haematology and Stem Cell Unit<br>ASL Latina, Italy
	Campus Bio-Medico University<br>Haematology and Stem Cell Transplant Unit<br>Rome, Italy
	ASL RM1<br>Haematology<br>Rome, Italy
	Sant'Eugenio Hospital, ASL Roma 2<br>Haematology<br>Rome, Italy
	S. Camillo Forlanini Hospital<br>Haematology and Stem Cell Transplant Unit<br>Rome, Italy

Table S2. Report example

Patient personal information, clinical data and biological material

Family name		Name		
Date of birth		Sex		
Ward / clinic / hospital		Physician		
Request ID		Report number		
Sample type		First pull	yes	no
Sample ml		Sample cellularity		
Date of collection		Date of cytometry study		
Diagnostic query and clinical information				

Quality control of the sample: cytometric myelogram

Erythroblasts<br>(CD138 neg / CD45 neg) = %	Lymphocytes<br>(SSC low /CD45++) = %	Myelocytes<br>(SSC high / CD45+) = %
Monocytes<br>(CD38/CD45/SSC intermediate) = %	Myeloid Precursors<br>(CD117/45+) = %	Mast Cells<br>(CD117+++ / SSC high) = %
Plasma cells (CD138/38++) = %	N° of total events acquired =	N° of total plasma cells acquired =

Analysis on plasma cell population (CD38/CD138 bright)

**Marker %**		**Marker**	**Cyto Ig Kappa %**	**Cyto Ig Lambda %**	***Ratio* kappa/lambda**
**CD38 = 100%++**		CD38 positive			
**CD138 = 100%++**		CD19 positive			
CD19 = %		CD19 negative			
CD20 = %		CD56 positive			
CD27 = %		CD56 negative			
CD28 = %		CD117 positive			
CD56 = %		CD19 positive / CD45 positive			
CD45 = %		CD19 positive / SAM positive			
CD81 = %		CD19 positive / SAM negative			
CD117 = %		CD19 negative / SAM positive			

SAM: Surface Aberrant Marker; Abnormal Kappa / Lambda = <0.5 o >4.0

Analysis on lymphoid population (SSC/CD45++)

**Marker %**		**Marker**	**Cyto Ig Kappa %**	**Cyto Ig Lambda %**	***Ratio* kappa/lambda**
CD19 = %		CD19 positive			
CD20 = %		CD19 positive / CD38 negative			
CD28 = %		CD19 positive / CD38 positive			
CD56 = %					
CD45 = %					
CD19/CD38 = %					

Final comment/conclusion

The plasma cell population - CD138^{+} and/or CD38^{+} - represents xx% of the entire cell population.

The analysis conducted on the PC population documents a xx% of clonal/pathological elements CDx^{pos}, CDxx^{neg}, restricted for the Kappa/Lambda Ig light chains flanked by CD19^{+}/CD45^{+} PCs with a normal Kappa/Lambda ratio. The B lymphoid population, which represents xx% of lymphocytes, has a normal/clonal Kappa/Lambda expression.

For MRD study: negative, positive, below the limit of quantification.

Unsuitable bone marrow sample: Hypocellular sample, possibly contaminated with peripheral blood due to low percentage of erythroblasts and/or myeloid precursors and mast cells and/or B-lymphoid precursors.
